# Annular Elastolytic Giant Cell Granuloma in a Post-COVID-19 Patient: A Case Report

**DOI:** 10.7759/cureus.114272

**Published:** 2026-08-10

**Authors:** Anna S Holcomb, Colleen M Emerson, Aleksandra Brown

**Affiliations:** 1 Transitional/Dermatology, Trinity Health, Ann Arbor, USA; 2 Dermatology, LewisGale Hospital Montgomery, Blacksburg, USA; 3 Dermatology, River Ridge Dermatology, Blacksburg, USA

**Keywords:** actinic granuloma, annular elastolytic giant cell granuloma, benign granulomatous disease, dermatological manifestation of covid-19 infection, granulomatous disease

## Abstract

Annular elastolytic giant cell granuloma (AEGCG) is a rare, benign granulomatous disease that most commonly affects middle-aged women with a history of extensive sun exposure. It typically presents as asymptomatic annular plaques with raised erythematous borders and hypopigmented, atrophic centers. Histologically, it is characterized by granulomatous infiltration with elastolysis and elastophagocytosis by multinucleated giant cells. Here, we present a case of a 69-year-old woman with a unique and widespread presentation of AEGCG to the trunk, legs, and arms following a recent history of COVID-19 infection.

## Introduction

Actinic granuloma is a rare, benign granulomatous disease that most commonly affects middle-aged women. Clinically, it presents as asymptomatic annular plaques with raised, inflammatory, erythematous borders with hypopigmented, atrophic centers in sun-exposed areas [1,2]. Histologically, lesions may demonstrate granulomatous infiltration with elastolysis and elastophagocytosis by multinucleated giant cells [3]. When clinical findings are present on non-sun-exposed skin, the disease is referred to as annular elastolytic giant cell granuloma (AEGCG) [2]. Elastophagocytosis is a characteristic of certain granulomatous disorders, such as AEGCG. It refers to the phagocytosis of elastic microfibers, which can be seen microscopically in the cytoplasm of histiocytes or multinucleated giant cells [4]. The exact pathogenesis of the disorder is unknown; however, it is hypothesized to involve an autoimmune response to elastic tissue damage. Although ultraviolet (UV)-induced elastic fiber damage is considered the primary trigger, immune dysregulation following viral infections has also been implicated in a variety of granulomatous and inflammatory dermatoses [5,6]. Notably, cutaneous lesions have been reported following COVID-19, and these findings may occur in the absence of respiratory symptoms or fever [7]. To our knowledge, reports of AEGCG developing following COVID-19 infection remain rare, making recognition of this potential association clinically relevant. We present a case of a patient with a recent history of COVID-19 who developed AEGCG involving sun-protected areas.

## Case presentation

A 69-year-old woman presented with a one-year history of an asymptomatic annular rash in the axilla that spread to the arms, legs, and the majority of the trunk during the winter months (Figures 1, 2). The patient denied systemic involvement and was initially treated empirically with fluconazole 200 mg once a week for three weeks due to concern for a superficial fungal infection given the presence of an annular rash. The patient did not improve following antifungal therapy. Laboratory studies drawn by the primary care physician were unremarkable. Punch biopsy was performed, which demonstrated a dermal palisading granulomatous reaction with elastophagocytosis and without an increase in interstitial mucin, consistent with actinic/annular elastolytic granuloma (Figures 3, 4). Although the rash began approximately one year before her presentation, the patient recalls having a recurrent COVID-19 infection around the time of the onset of the rash. She also reports increased emotional stress prior to rash expansion. The patient was counseled on treatment options and was scheduled to initiate hydroxychloroquine, but ultimately declined treatment with hydroxychloroquine due to the side effect profile. On follow-up evaluation one month later, the patient reported worsening of the rash with new involvement of her legs and back. She was prescribed triamcinolone 0.1% cream at that time, but has since been lost to follow-up (Figures 5, 6).

**Figure 1:**
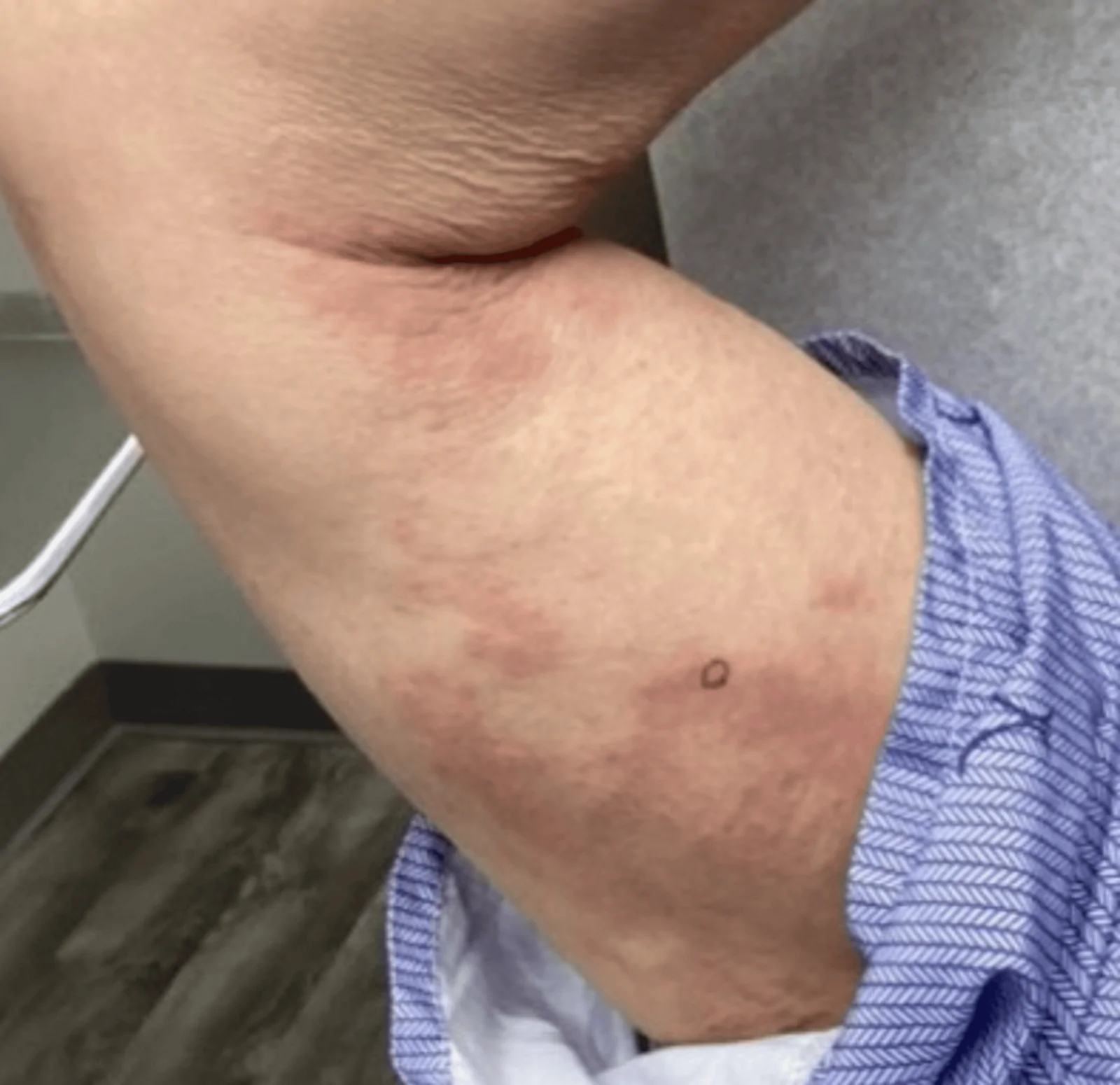
Patient’s initial presentation, physical exam demonstrating annular rash to the axilla.

**Figure 2:**
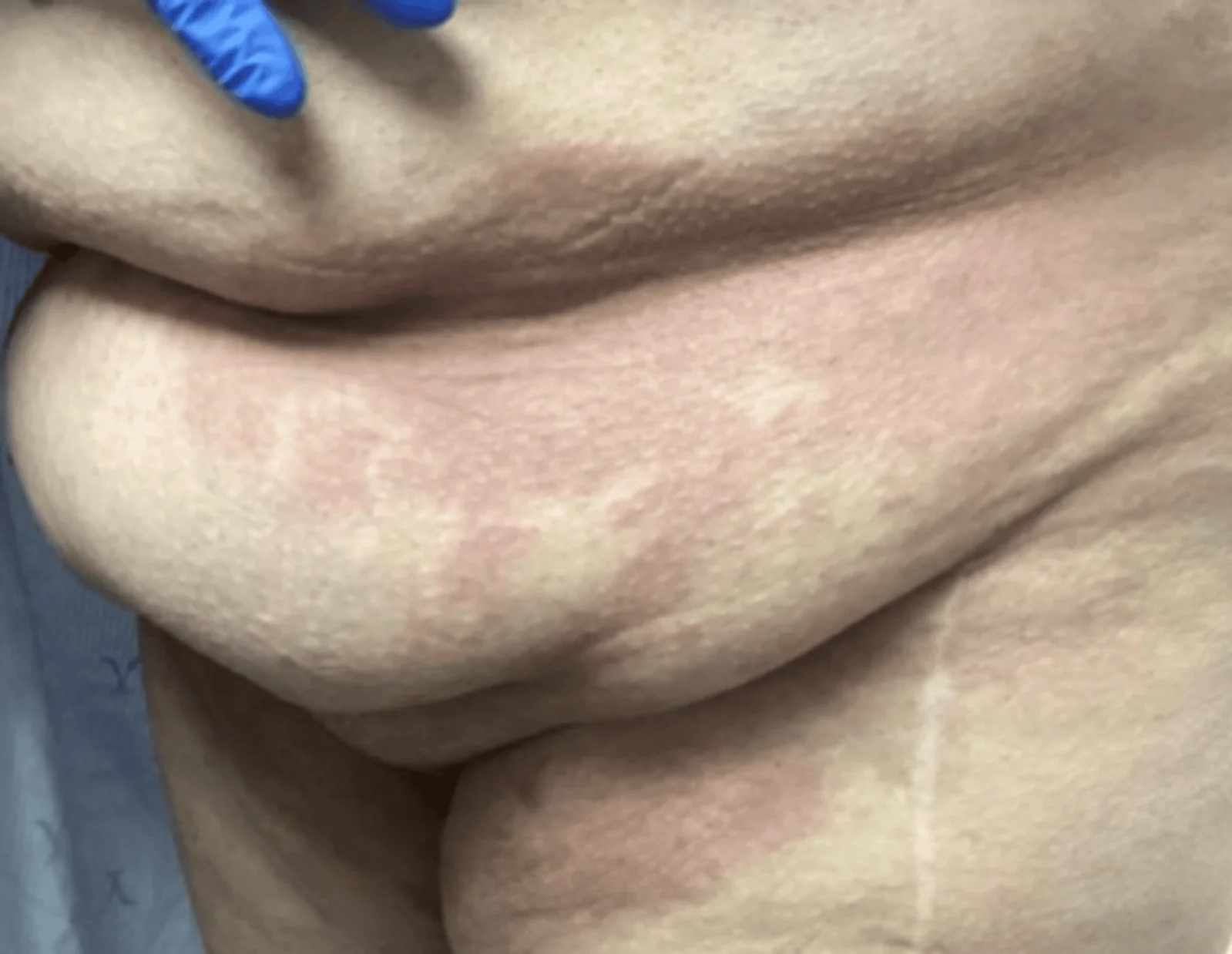
Patient’s initial presentation, physical exam demonstrating annular rash to the trunk.

**Figure 3:**
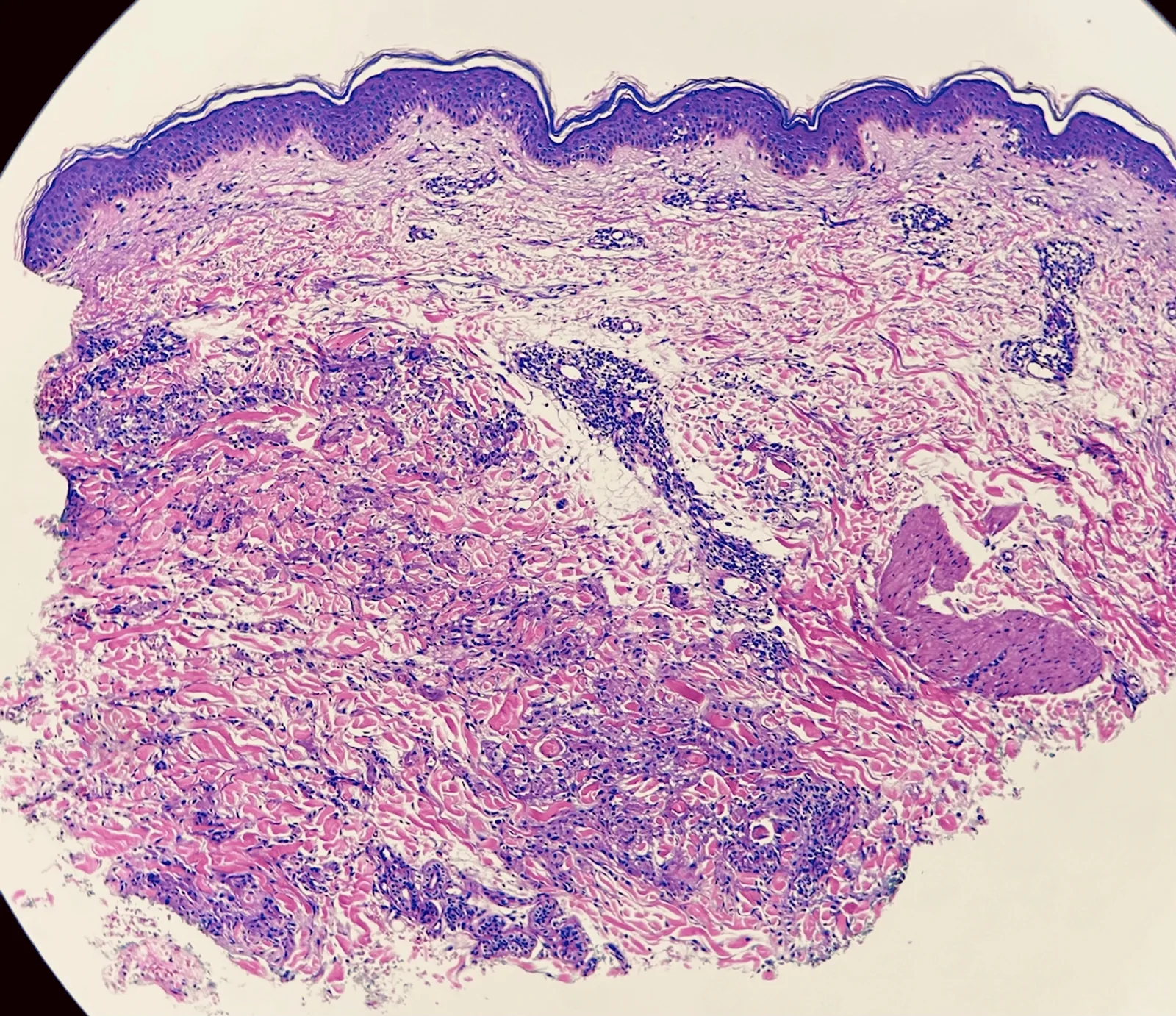
Shave biopsy of sun-damaged skin. Sun-damaged skin containing a portion of dermal palisaded granulomatous reaction. No obvious increase in interstitial mucin is seen. In the clinical context, these histologic findings are consistent with a diagnosis of annular elastolytic giant cell granuloma or actinic granuloma (hematoxylin and eosin stain, original magnification x4).

**Figure 4:**
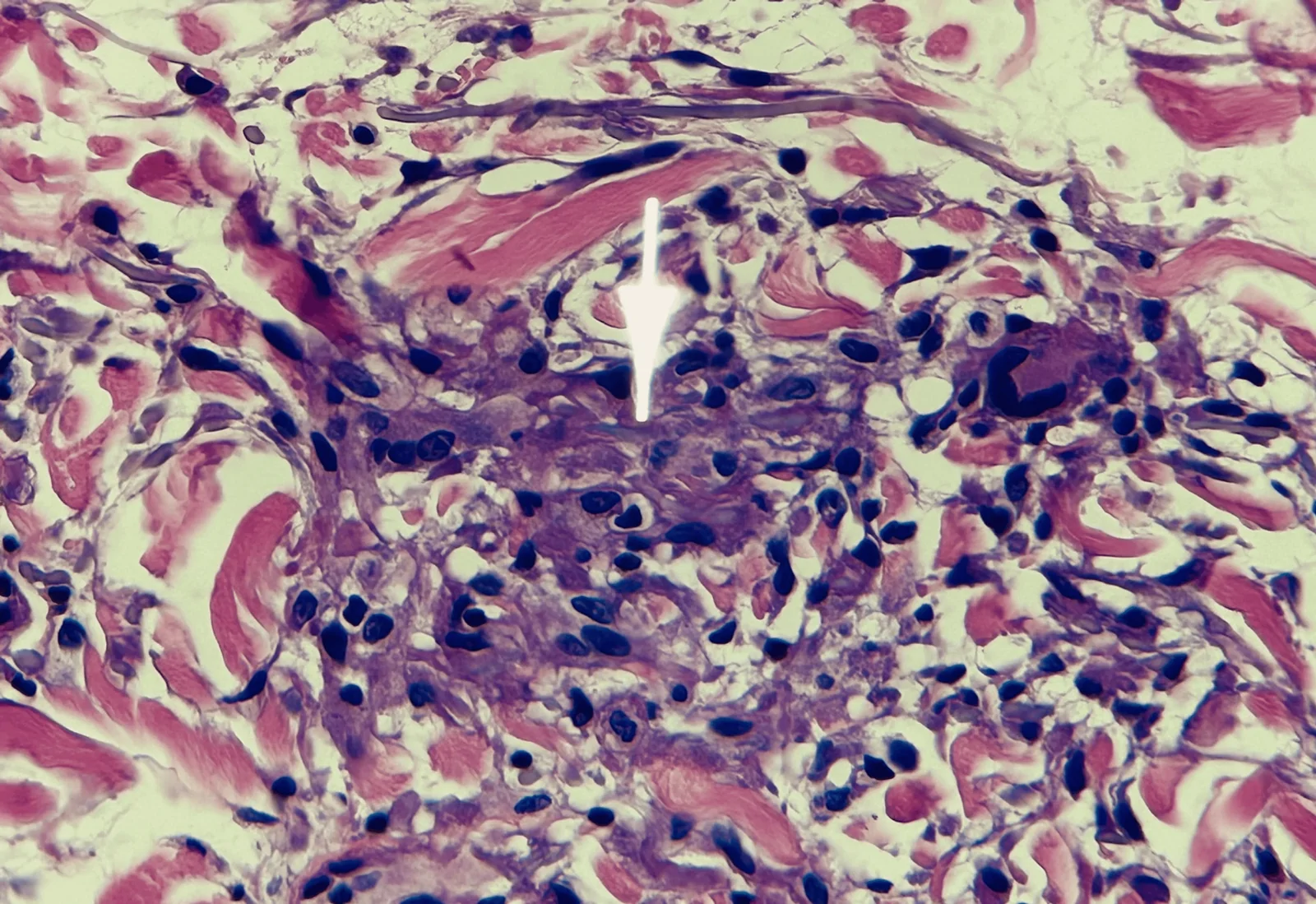
Shave of sun-damaged skin. Shave of sun-damaged skin containing a portion of a dermal palisaded granulomatous reaction. No obvious increase in interstitial mucin. Arrow demonstrating elastophagocytosis (hematoxylin and eosin stain, magnification x40).

**Figure 5:**
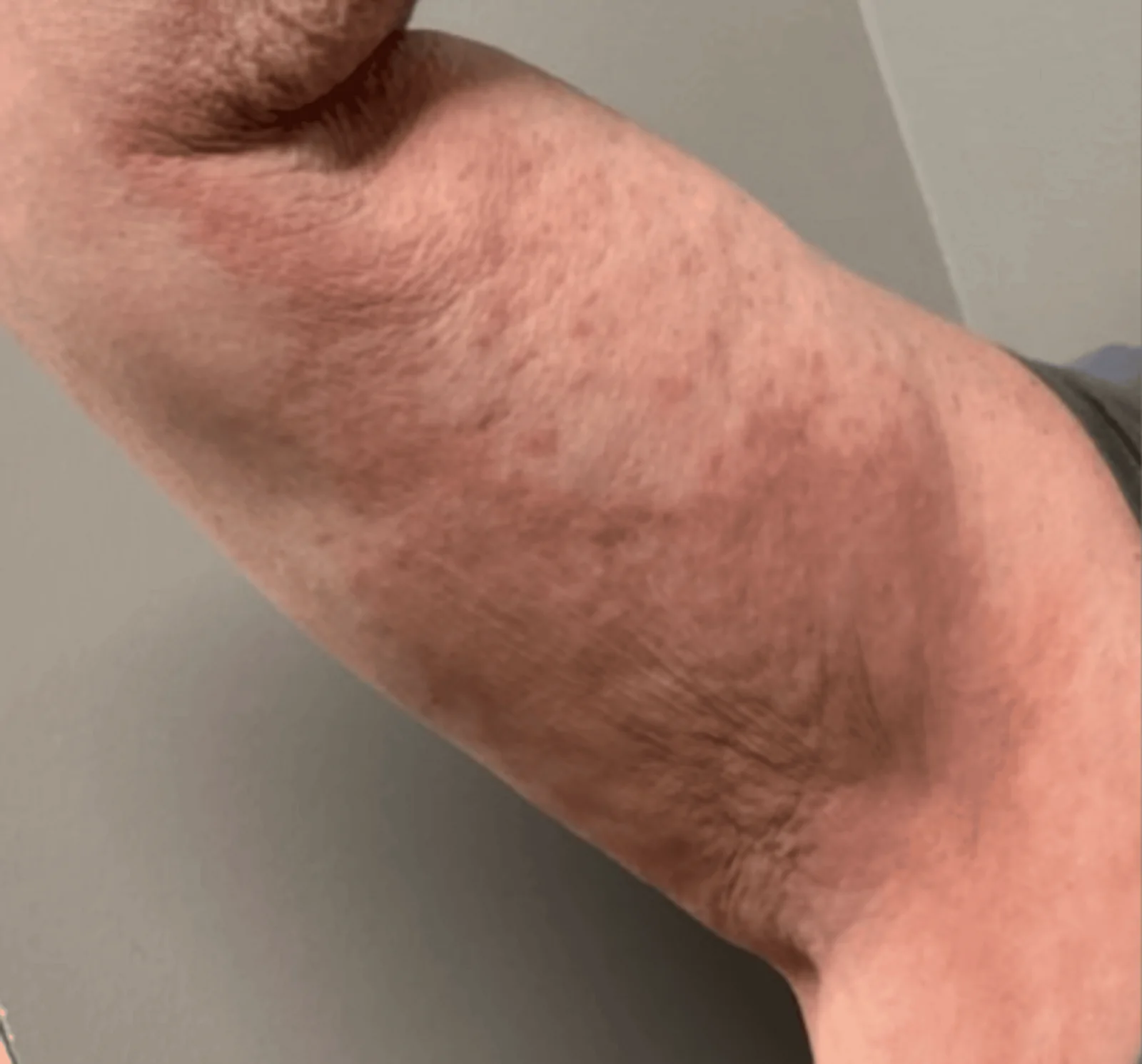
Patient’s presentation on follow-up evaluation, demonstrating expansion of annular rash on the extremity.

**Figure 6:**
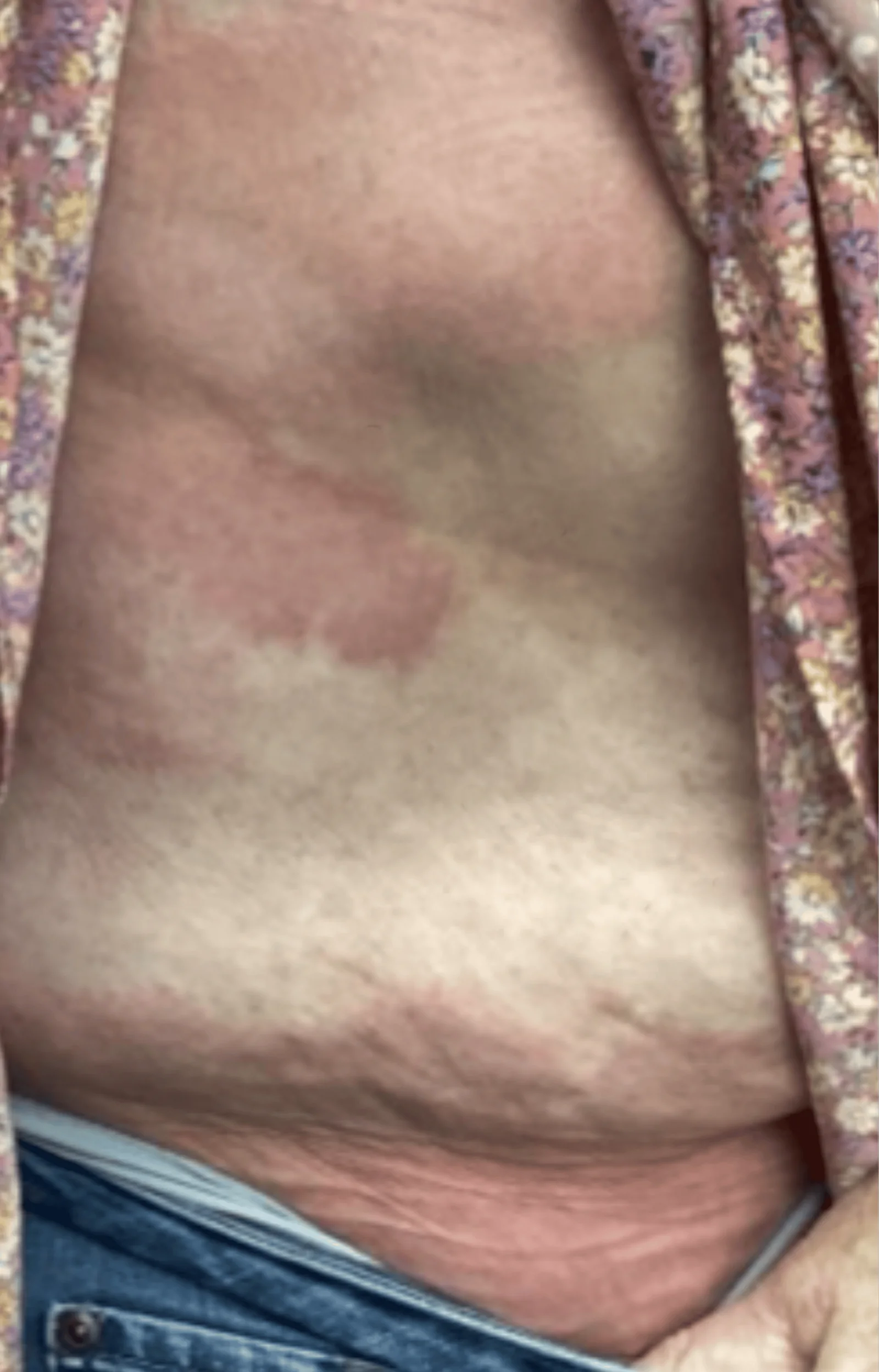
Patient’s presentation on follow-up evaluation, demonstrating expansion of annular rash on the trunk.

## Discussion

AEGCG is a rare, benign granulomatous disease that predominantly affects middle-aged women with a history of excessive sun exposure. This disorder was first described by O’Brien in 1975 as ring-shaped inflammatory papules and plaques in sun-damaged, “elastotic” skin [5,8].

AEGCG is a persistent condition that is difficult to treat and often exhibits a poor response to treatment [9]. The differential diagnosis for AEGCG includes granuloma annulare, tinea corporis, sarcoidosis, lupus erythematosus, and other granulomatous diseases. As discussed previously, the histopathological findings are the mainstay of diagnosis [2]. Several treatment options exist, including topical, intralesional, and systemic corticosteroids; hydroxychloroquine; cyclosporine; cryotherapy; methotrexate; isotretinoin; acitretin; pentoxifylline; pulse-dye laser; fractional carbon dioxide laser; and retinoid therapy combined with psoralen plus UV A (PUVA) [8].

AEGCG is thought to result from inflammation secondary to extensive actinic damage [9]. Solar UV exposure is considered the primary initiating factor, selectively injuring elastic fibers within the upper and mid-dermis. The resulting altered elastic tissue is thought to act as an antigenic stimulus, provoking a predominantly CD4+ T-cell-mediated immune response aimed at tissue repair. However, this process ultimately becomes dysregulated, culminating in granulomatous inflammation rather than effective regeneration [10].

The patient’s case is notable due to the extent of skin involvement. AEGCG often presents with small, coalescing papules, generally involving fewer than 10 lesions and measuring less than 10 cm in diameter [9]. In contrast, this patient presented with numerous, large, coalescing plaques distributed diffusely throughout the trunk and extremities.

Numerous cutaneous eruptions have been observed following an infection with COVID-19, the most common of which are chilblain-like, maculopapular, vesicular, urticarial, and livedoid [11]. Although this patient experienced cutaneous manifestations following a COVID-19 infection, her presentation of AEGCG differs from the current literature. However, a recent case report described a 62-year-old farmer who experienced widespread AEGCG in both sun-exposed and protected areas shortly after receiving the COVID-19 viral vector vaccine. Similar to our patient, the reported workup, including hematological, coagulation, protein, lipid, and renal profiles, was unremarkable [12].

While SARS-CoV-2 can induce a hyper-inflammatory response and a subset of patients experience cutaneous manifestations following viral infection or treatment, it remains unclear whether an association exists between COVID-19 infection or viral vector vaccine and AEGCG [13]. This case presents an interesting opportunity for further research.

## Conclusions

This patient’s clinical presentation, including extensive skin involvement and a recent COVID-19 infection, represents an atypical case of AEGCG. This case serves as a point of reference for physicians to consider AEGCG within their differential for patients presenting with granulomatous-type lesions.

## References

[1] Coutinho ID, Ramos LI, Brites MM, Tellechea O (2015). O'Brien actinic granuloma: a case report and brief review of literature. Indian J Dermatol.

[2] Liu W, Ma DL (2019). Actinic granuloma. CMAJ.

[3] Limas C (2004). The spectrum of primary cutaneous elastolytic granulomas and their distinction from granuloma annulare: a clinicopathological analysis. Histopathology.

[4] El-Khoury J, Kurban M, Abbas O (2014). Elastophagocytosis: underlying mechanisms and associated cutaneous entities. J Am Acad Dermatol.

[5] O'Brien JP (1975). Actinic granuloma. An annular connective tissue disorder affecting sun- and heat-damaged (elastotic) skin. Arch Dermatol.

[6] Gutiérrez-González E, Pereiro M Jr, Toribio J (2015). Elastolytic actinic giant cell granuloma. Dermatol Clin.

[7] Wang CJ, Worswick S (2021). Cutaneous manifestations of COVID-19. Dermatol Online J.

[8] Promsena P, Triyangkulsri K, Rutnin S (2023). Two case reports of elastolytic giant cell granuloma on the palms. Clin Cosmet Investig Dermatol.

[9] Rosenbach MA, Wanat KA (2024). Non-infectious granulomas. Dermatology.

[10] Mistry AM, Patel R, Mistry M, Menon V (2020). Annular elastolytic giant cell granuloma. Cureus.

[11] Panda M, Dash S, Behera B, Sil A (2021). Dermatological manifestations associated with COVID-19 infection. Indian J Dermatol.

[12] Soares-Neto RF, Ramalho AR, Oliveira PD, Araújo JM, Tavares MA, Martins-Filho PR, Portugal FM (2024). Annular elastolytic giant cell granuloma following COVID-19 viral vector vaccine: first case report. J Dermatol.

[13] Merad M, Martin JC (2020). Pathological inflammation in patients with COVID-19: a key role for monocytes and macrophages. Nat Rev Immunol.

